# Developmental Cues and Molecular Drivers in Myelinogenesis: Revisiting Early Life to Re-Evaluate the Integrity of CNS Myelin

**DOI:** 10.3390/cimb44070222

**Published:** 2022-07-19

**Authors:** Iasonas Dermitzakis, Maria Eleni Manthou, Soultana Meditskou, Dimosthenis Miliaras, Evangelia Kesidou, Marina Boziki, Steven Petratos, Nikolaos Grigoriadis, Paschalis Theotokis

**Affiliations:** 1 Department of Histology-Embryology, School of Medicine, Aristotle University of Thessaloniki, 54124 Thessaloniki, Greece; iasonasd@auth.gr (I.D.); mmanthou@auth.gr (M.E.M.); sefthym@auth.gr (S.M.); miliaras@auth.gr (D.M.); 2 Laboratory of Experimental Neurology and Neuroimmunology, Second Department of Neurology, AHEPA University Hospital, 54621 Thessaloniki, Greece; bioevangelia@yahoo.gr (E.K.); bozikim@auth.gr (M.B.); ngrigoriadis@auth.gr (N.G.); 3 Department of Neuroscience, Central Clinical School, Monash University, Prahran, VIC 3004, Australia; steven.petratos@monash.edu

**Keywords:** oligodendrogenesis, myelinogenesis, myelin formation, embryology, CNS development, neural tube development, morphogen signaling

## Abstract

The mammalian central nervous system (CNS) coordinates its communication through saltatory conduction, facilitated by myelin-forming oligodendrocytes (OLs). Despite the fact that neurogenesis from stem cell niches has caught the majority of attention in recent years, oligodendrogenesis and, more specifically, the molecular underpinnings behind OL-dependent myelinogenesis, remain largely unknown. In this comprehensive review, we determine the developmental cues and molecular drivers which regulate normal myelination both at the prenatal and postnatal periods. We have indexed the individual stages of myelinogenesis sequentially; from the initiation of oligodendrocyte precursor cells, including migration and proliferation, to first contact with the axon that enlists positive and negative regulators for myelination, until the ultimate maintenance of the axon ensheathment and myelin growth. Here, we highlight multiple developmental pathways that are key to successful myelin formation and define the molecular pathways that can potentially be targets for pharmacological interventions in a variety of neurological disorders that exhibit demyelination.

## 1. Introduction

As the regulator of all cognitive, sensory, and motor activity, the nervous system is the most complex biological system in humans; the complexities of integrated neural networks are a hot area of intensive research that will require multidisciplinary investigations to address a variable array of neurological disorders that remain an unmet medical need. The main types of cells in the nervous system are neurons and glial cells with the latter performing vital supporting roles [1,2]. The glial/neuronal ratio differs uniformly across brain regions of mammalian species, underlining the pivotal role of interaction between glial cells and neurons for appropriate integration of the central nervous system (CNS) to coordinate neurophysiological and cognitive functions [3]. For propagation of action potentials to ensue in neurons, axonal myelination is crucial [4,5]. The cells responsible for myelination are the oligodendrocytes (OLs) in the CNS and Schwann cells in the peripheral nervous system (PNS). Rudolf Virchow initially designated the term “myelin” in 1854, named after the Greek word “marrow” (myelos), since it is especially plentiful in the brain’s center, or marrow [6]. He posited that neurons produced myelin, but Pío del Río Hortega’s better histological staining processes almost a century ago revealed that myelin is created by specific glial cells, which are OLs [7,8].

The CNS macroglia and neurons have a common embryonic origin from the neuroectoderm, most prominently from neuroepithelial cells of the telencephalic ventricular and subventricular zone (VZ and SVZ), while the spinal cord is supplied with cell derivatives exclusively from the central canal [9,10]. Newborn CNS is radically unmyelinated with a sparse developing pool of unipotent cells, namely oligodendrocyte precursor cells (OPCs), following their birth with gradual widespread functionality in the first few years of childhood [11]. Myelination persists in an asynchronous spatiotemporal pattern through adolescence towards adulthood, coinciding with the establishment and maintenance of correct circuit function and cognitive development [12,13]. Mature myelin sheaths remain stable by and large; however, they maintain the capacity to remodel and reorganize if need be [14]. As expected, aging promulgates limit resources and energy deficiency to sustain such developmental processes, thus cellular senescence is a common event [15]. Consequently, there is a variety in the patterns of myelination, with qualitative- and quantitative-ontogenic checkpoints, throughout human life.

In this review, we focus on the de novo synthesis of myelin referred to herein as myelinogenesis. This is the primordial pattern of myelination, which starts prenatally and predominates during the first two years of human life [16]. In order for myelinogenesis to happen, neural stem cells (NSCs) need to undergo specific developmental stages, with the process of oligodendrogenesis, as well as additional steps for the maintenance of these primary myelin sheaths. Interestingly, it is possible that lifelong myelinogenesis may still occur in specific CNS regions through quiescent, adult OPCs (aOPCs), based on miscellaneous factors, such as unmyelinated space, new OLs turnover, energy balance, and neural circuit activity [17]. Such processes are broadly defined as adaptive myelination or myelin remodelling/plasticity which is under fine regulation and is generally restricted. Lastly, another crucial factor that may trigger myelinogenesis is injury and disease, such as demyelination, and is discussed briefly towards the end, with a process known as remyelination.

## 2. Myelinogenesis and Myelin Development: A Spatiotemporal Coordination

### 2.1. Primordium Regions of OPCs

**Figure 1:**
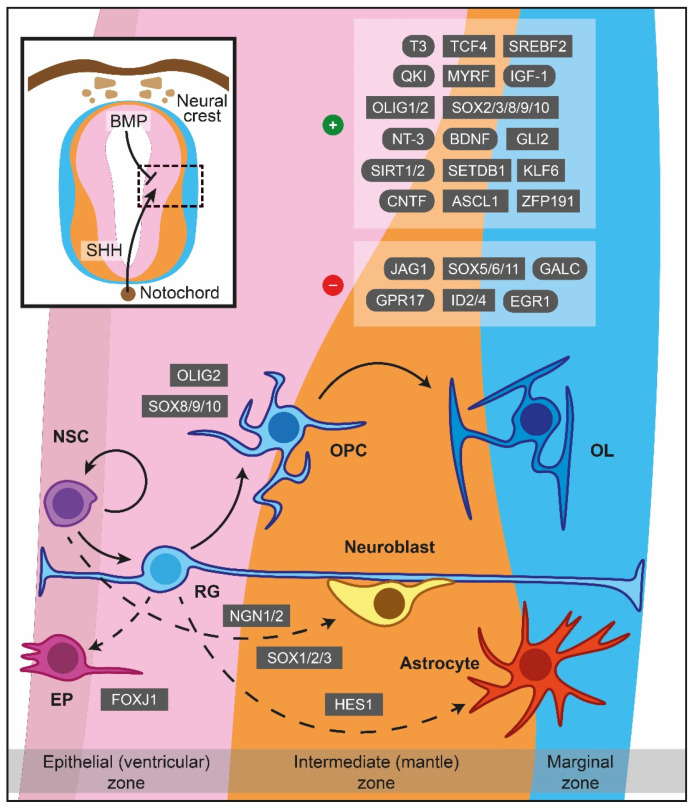
Major cues of OPCs generation and differentiation during myelinogenesis in the prenatal period. In the neuroepithelium lining the neural tube, NSCs are under the influence of notochord-derived SHH, which drives the cells to become OPCs through OLIG2, SOX8/9/10 or follow neuronal fate (neuroblasts) via NGN1/2 and SOX1/2/3. BMP originated from the neural crest instructing NSCs to become astrocytes, controlled by HES1 as well. FOXJ1 is a crucial transcription factor for the ependymal trajectory. The positive and negative cues controlling OPCs differentiation are displayed in the upper right boxes. The hatched box depicts a representative area around sulcus limitans (between alar and basal plates). Dashed lines showcase naturally occurring processes, albeit not addressed in detail in the current review. ASCL1: Achaete-scute family bHLH transcription factor 1, BDNF: Brain-derived neurotrophic factor, BMP: Bone morphogenetic protein, CNTF: Ciliary neurotrophic factor, EGR1: Early growth response 1, EP: Ependymal cells, FOXJ1: Transcription factor forkhead box J1, GALC: Galactosylceramidase, GLI2: Glioma-associated oncogene family zinc finger 2, GPR17: G protein-coupled receptor 17, HES1: Hes family bHLH transcription factor 1, ID2: Inhibitor of DNA binding 2, ID4: Inhibitor of DNA binding 2, IGF-1: Insulin-like growth factor 1, JAG1: Jagged canonical Notch ligand 1, KLF6: Kruppel-like factor 6, MYRF: Myelin regulatory factor, NGN1: Neurogenin-1, NGN2: Neurogenin-2, NSC: Neural stem cells, NT-3: Neurotrophin 3, OL: Oligodendrocytes, OLIG1: Oligodendrocyte transcription factor 1, OLIG2: Oligodendrocyte transcription factor 2, OPC: Oligodendrocyte precursor cell, QKI: Quaking homolog, KH domain RNA binding, RG: Radial glia, SET domain bifurcated histone lysine methyltransferase 1, SETDB1: SHH: Sonic hedgehog signaling molecule, SIRT1: Sirtuin 1, SIRT2: Sirtuin 2, SOX: Sex-determining region Y-box transcription factor, SREBF2: Sterol regulatory element-binding transcription factor 2, T3: Triiodothyronine, TCF4: Transcription factor 4, ZFP191: Zinc finger protein 191.

The mammalian CNS emerges from an ectodermal, neuroepithelial lining of the neural tube in the developing embryo [9,18]. Multiple divisions give rise to radial glia (RG), a multipotent neural stem population that colonizes the newly-formed ventricular walls (Figure 1) [18,19]. The VZ is the primary embryonic site for OPCs production through asymmetrical division of the RG cells. In mice, OPCs are firstly detected in the ventral VZ closer to the floor plate on embryonic day 12.5 (E12.5), and in humans at gestational week (GW) 6.5 (~E45) [20]. More specifically, the outer SVZ (oSVZ) is an enlarged cortical germinal zone only generated in humans [21]. In oSVZ, a distinct RG cell population termed as outer RG (oRG) is located peripherally and gives rise to a transit-amplifying population, which is an additional source of OPCs supplying the human cortex [19,21]. 

In the human forebrain, the first wave of OPCs originates from the medial ganglionic eminence (MGE) and the anterior entopeduncular area (AEP), while a second batch emerges postnatally from the lateral or caudal ganglionic eminences, establishing a sufficient amount of OPCs in the cerebral cortex [22]. OPCs of the human forebrain appear in the SVZ of the MGE at GW7.5, whilst at E12 in mice [19]. In the spinal cord, the majority of the nascent OPCs (about 80% of the total number) complete their formation at the motor neuron progenitor (pMN) domain of the ventral spinal cord, while the pool is enriched later at E15.5 by additional OPCs migrating from dorsal regions [23]. Lastly, the cerebellar OPCs are derived from the metencephalic ventral rhombomere 1 region, manifesting their presence at E16.5 and are reinforced additionally with a secondary population originating from the cerebellar VZ [10].

### 2.2. Molecular Signals Driving Myelinogenesis

As has been articulated from the experimental evidence, the inauguration of myelinogenesis necessitates the formation of OPCs from multipotent NSCs, which ultimately give rise to mature myelinating OLs through a multistep process (Figure 1) [18,24]. A vital step herein lies in OPCs’ ability to migrate toward miscellaneous sites and proliferate, based predominately on environmental stimuli. These cells become post-mitotic, exiting the cell cycle to express a substantial amount of myelin-associated proteins and differentiate into mature pre-myelinating OLs [23]. Following the proper recognition, targeting and ensheathing specific nerve fibers is the subsequent critical milestone where each pioneer process creates lamellar extensions that stretch and elaborate circumferentially around the target axon [24]. As a new membrane is generated at the leading edge of the forming myelin sheath’s inner tongue, which starts to resemble a spiral cross-sectional shape, the sheath continues to spread along the axonal length. The secured stability and maintenance of a newly-formed myelin sheath is the concluding event. Specific developmental cues and molecular drivers regulate all the aforementioned cellular activities and are enlisted in full capacity in Table A1, Table A2 and Table A3.

#### 2.2.1. Formation of OPCs

OPCs being generated from the ventral VZ are under the influence of the morphogen molecule Sonic hedgehog (SHH) secreted from the notochord, while the dorsal counterparts are SHH-independent [25,26]. SHH signalling drives NSCs into a neuronal or OLs lineage fate superseding the effect of bone morphogenetic proteins (BMPs) which favour astroglial generation (Figure 1) [27,28]. Early secretion of SHH promotes motor neuron lineage formation, while interaction in later time periods promotes OLs differentiation [29]. Interestingly, the concentration of SHH can be controlled by sulfatase 1 expression in the ventral neuroepithelium prior to OPCs specification [30], whereas fibroblast growth factor (FGF) signalling is of paramount importance for further OLs differentiation, especially in the spinal cord [31,32]. 

Oligodendrocyte transcription factor 2 (OLIG2) is the primary regulator of OPCs generation [33,34], and its gene expression can be potentially repressed throughout the pre to postnatal period by paired box 6 (PAX6), Brahma-related gene-1 (BRG1), Iroquois homeobox 3 (IRX3), histone deacetylase (HDAC) 1, HDAC2, Distal-less homeobox (DLX) 1 and DLX 2 [35,36,37,38,39,40,41]. On the other hand, oligodendrocyte transcription factor 1 (OLIG1) is activated in later stages of OLs development [42]. Interestingly, the Hes family bHLH transcription factor (HES1) can drive RG to an astrocytic phenotype [43], while co-occurrence of OLIG2 with neurogenin-1 or neurogenin-2 supports motor neuron production [38,44,45]. 

Members of the sex-determining region Y-box transcription factor (SOX) family, such as SOX1, SOX2, and SOX3, can also direct OPCs towards a neuronal fate [33], in contrast to SOX8, SOX9, and SOX10, which favour the turnover of NSCs to OPCs in an autonomous manner [34,35,36]. Additionally, transcription factor forkhead box J1 (FOXJ1) supports the retention of RGs as ependymal cells throughout ventricles. Lastly, glioma-associated oncogene family zinc finger 2 (GLI2), myelin transcription factor 1 (MYT1), NK2 homeobox 6 (NKX2-6), and chromodomain-helicase-DNA-binding protein 8 (CHD8), among others (Table A1), are embryonic cues for OLs specification that vary within CNS regions indicating brain region specificity [37,38,39,40].

#### 2.2.2. Migration

SHH presence is equally catalytic to OPCs migration [41]. Platelet-derived growth factor subunit A (PDGFA) and its cognate receptor, PDGF receptor alpha (PDGFRα), are essential positive drivers for OPCs migration [42]. In line with this, SOX5, SOX6, SOX9, and SOX10 stimulate the migration, ensuring PDGF responsiveness [43,44]. Chondroitin sulfate proteoglycan neuron-glia antigen 2 (NG2) and ephrin-B2/B3 molecules control OPCs polarity and contact abilities, promoting or intercepting migration, respectively [45,46]. Nestin, neural cell adhesion molecule (NCAM), and OLIG1 can also act as chemoattractants, determining cytoskeletal plasticity as well as OPCs motility [47,48,49,50,51]. Other migration chemoattractants are 2′,3′-cyclic nucleotide 3′ phosphodiesterase (CNPase), OLIG2, hepatocyte growth factor (HGF), thrombospondin 1, endothelin 1 (ET-1), oligodendrocyte specific protein (OSP), OSP–associated protein (OAP-1), N-cadherin (NCAD), merosin, fibronectin, and integrin subunit beta 1 (αvβ1 integrin) [52,53,54,55,56,57,58,59,60]. Spassky et al. suggested that netrin-1 is a candidate mediator for chemoattraction during migration [61]. However, other studies considered this molecule as a chemorepellent, antagonizing PDGF [62,63]. 

More growth factors and associated molecules, such as vascular endothelial growth factor A (VEGF-A) combined with VEFG receptor 2 (VEGFR2), can act as chemoattractant molecules for OPCs migration along with miscellaneous members of the transforming growth factor beta (TGF-β) family (e.g., BMP7 and BMP4), and Gαi-linked sphingosine-1-phosphate receptor (S1PR) 1 and S1PR3 [64,65,66]. In contrast to these specific sphingosine molecules, S1PR2 and S1PR5 negatively regulated migration [66]. Moreover, although C-X-C motif chemokine receptor (CXCR) 4, C-X-C motif chemokine ligand (CXCL) 12 and semaphorin 3F have chemoattractive effects on the OPCs migration, semaphorin 3A, CXCL1, and CXCR2 inhibit migration [61,67,68]. In addition, tenascin-c inhibited OPCs migration, whilst both claudin (CLDN) 1 and CLDN3 supported OPCs relocation, validated also in human specimens [59,69,70]. 

#### 2.2.3. Proliferation

Specific driver molecules that participate in migration, such as PDGFA and PDGFRα, contribute additionally to the OPC proliferation [47,71]. Interestingly, in the spinal cord, the mitogenic effect of PDGF was enhanced by chemokine CXCL1 and CXCR2 [44,72], while CXCL12 had a proliferative effect on OPCs, mediated by its receptor CXCR4 [73]. More growth factors, such as FGF2, brain-derived neurotrophic factor (BDNF), and epidermal growth factor (EGF) are shown to play a vital role in OPCs proliferation [74,75,76]. 

Associate developmental pathways are also implicated in this step; PDGF-mediated proliferation depends largely on Wnt/β-catenin and PI3K/AKT/mTOR pathways [77,78]. Furthermore, jagged canonical Notch ligand 1 (JAG1) promotes OPCs proliferation and critically blocks the subsequent differentiation step [79]. Carrying on subcellular, CHD7 and CHD8 regulate gene expression in specific brain regions [80,81]. Another member of the SOX family, SOX9, supports the development of OLs in the cerebellum, regulating the timing of proliferation [82]. MYT1, NCAM, cyclin-dependent kinase inhibitor 1B (p27^KIP1^), oligodendrocyte myelin glycoprotein (OMgp), and tubulin polymerization promoting protein (TPPP) are negative regulator cues for OPCs proliferation [83,84,85,86,87]. Interestingly, overexpression of inhibitor of DNA binding (ID) 2 and ID4 enhances proliferation [88,89]. Similarly, expression of SHH, HGF, neurotrophin-4 (NT-4), noggin, superoxide dismutase 1 (SOD1), neurotrophin-3 (NT-3), achaete-scute family bHLH transcription factor 1 (ASCL1), PAX6, CLDN1, and CLDN3 promotes the proliferation process [41,54,70,90,91,92,93,94,95].

Integrin-mediated signalling and, more specifically, OSP, OAP-1, αvβ1 integrin, αvβ3 integrin, fibronectin and laminin are pivotal mediators in cytoskeletal remodelling of proliferating OPCs [56,96,97]. Gadea et al. revealed that ET-1 is a candidate molecule for enhancing cell migration without influencing proliferation [60]. Later, Adams and colleagues underscored that loss of ET-1 reduces OPCs proliferation in the developing SVZ via directly binding to endothelin type B receptor (ETBR) [98]. A reduced OPCs proliferation is observed in *GS homeobox 1/2* (*Gsx1/2*) mutant embryos, whereas galectin-4 (GAL-4) treatment increased the proliferation [99,100]. At last, NRG1 and SOX2 induce cell division [101,102]; however, the latest data demonstrate that NRG1 acting via ErbB did not alter the proliferation state of OPCs [103]. 

#### 2.2.4. Differentiation

OLIG1 and OLIG2 are heavily involved in the post-proliferating step of myelinogenesis, defining the initiation of OPCs differentiation (Figure 1) [51,53,104], while BMPs seem to inhibit this process by downregulating myelin protein expression [105]. The effect can be reversed by using a physiological antagonist of BMP4, such as noggin, which may restore differentiation [91,106,107]. OLIG2 appears to interact with a variety of factors, such as ASCL1, BRG1, transcription factor 4 (TCF4), and SET domain bifurcated histone lysine methyltransferase 1 (SETDB1) to ensure proper OPCs differentiation [108,109,110,111,112]. G protein-coupled receptor 17 (GPR17) can act as a downregulator of OLIG1 that negatively controls the maturation and coordinates the generation of myelinating OLs from pre-myelinating OLs through ID proteins [113]. Although overexpression of ID2 and ID4 both regulate myelin gene expression by inhibiting OLs differentiation [89,114], they are not the major in vivo repressors of differentiation [115]. Moreover, decreased levels of OLIG1 and myelin regulatory factor (MYRF) were observed under early growth response 1 (EGR1) and SOX11 overexpression, delineating the inhibitory action of the latest in OPCs differentiation [116,117]. Intriguingly, MYRF is a unique regulator participating in the late stages of OLs maturation and myelination, while the action of the other OLs’ lineage transcription factors is restricted on OPCs specification or initial differentiation of OLs [118].

SOX family proteins are also participating in the OLs differentiation. In particular, SOX2 and SOX3, through negative regulation of miR145, promote OLs maturation [119], while SOX5 and SOX6 increase PDGFRα expression, maintaining OLs in their immature state [44]. For the terminal differentiation of OLs, SOX8, SOX9, and SOX10 are required [82,120,121,122]. The state of myelinogenesis-associated gene expression is uniformly affected by NKX2-2 and NKX2-6 [40,123,124]. Ji et al. suggested a mechanism regarding NKX2-2-mediated inhibition of OLs differentiation via regulation of sirtuin 2 (SIRT2), which generally is a positive cue for OLs maturation [125]. Similarly, sirtuin 1 (SIRT1) participates in the differentiation of OPCs during development [126] through cytoskeleton-related OLs proteins. The Kruppel-like factor 6 (KLF6) is another transcription factor promoting OPCs differentiation through glycoprotein 130 (GP130)-signal transducer and transcription activator 3 (STAT3) signalling [127]. Growth factor-wise, BDNF is a regulator of OLs differentiation operating via binding to tyrosine receptor kinase B (TrkB) and enhancing the MAPK pathway to upregulate gene expression during OLs maturation [75,77,128]. Evidently, NT-3 is important for the transition of immature OLs to myelin-forming cells by recruiting c-Fos protein-activating protein kinase C (PKC) and tyrosine kinase activities [129,130]. Insulin-like growth factor 1 (IGF-1) is another main factor in assisting the development of OPCs to mature OLs [131]. In accordance with that, GRB2 associated binding protein 1 (GAB1) absence decreased OLs differentiation, acting as a novel target of PDGF [132]. Incidentally, Canoll et al. suggested that NRG1 is a negative regulator of OPCs differentiation [101], while Brinkmann et al. later demonstrated that NRG1 is required for OPCs differentiation [103].

As far as metabolism is concerned, quaking homolog, KH domain RNA binding (QKI)-5 forms a complex with sterol regulatory element-binding transcription factor 2 (SREBF2) that regulates the transcription of genes responsible for cholesterol biosynthesis in OLs during differentiation [133]. Lack of transactive response DNA-binding protein 43 (TDP-43) results in lower SREBF2 and low-density lipoprotein receptor (LDLR) expression and cholesterol levels in vitro and in vivo, indicating the potential role of TDP-43 in cholesterol homeostasis in OLs, which is linked with the proper completion of OLs development [134]. In the same manner, ectonucleotide pyrophosphatase/phosphodiesterase 6 (ENPP6) participates in OLs maturation via a supplement of OLs with choline [135]. Most importantly, triiodothyronine (T3) is a key molecule for blocking OPCs proliferation and promoting their differentiation into mature OLs [136,137]. Thyroid hormone receptor alpha (TRα) is found both in OPCs and mature OLs, whilst thyroid hormone receptor isoform beta 1 (TRβ1) is located only in mature OLs [138]. The OPCs differentiation is mediated by the TRα, while TRβ1 is responsible for promoting myelinogenesis in later stages [77]. Overexpression of HES5 decreases the levels of TRβ1 receptors, while ASCL1 increases them, demonstrating their role in regulating OLs differentiation timing [139]. The neurogenic locus notch homolog protein 1 (NOTCH1) is another receptor that also regulates the differentiation timing [140]. Interestingly, JAG1 is a receptor’s ligand responsible for inhibiting OLs differentiation, while contactin 1 (CNTN1) is another ligand with the opposite function [79,141].

Other membrane molecules which repress OPCs differentiation are NCAM and leucine-rich repeat, and Ig-like domain-containing Nogo receptor interacting protein 1 (LINGO-1) [142,143]. OLs maturation is negatively affected by GAL-4 and galactosylceramidase (GALC), while prominin-1, GLI2, p21-activated kinase 1 (PAK1), myelin-associated glycoprotein (MAG), SOD1, ciliary neurotrophic factor (CNTF), and inward rectifying potassium channel 4.1 (Kir4.1) are crucial for proper differentiation [38,95,100,144,145,146,147,148,149]. On the other hand, proper completion of OLs differentiation requires zinc finger protein 191 (ZFP191) [150]. Microtubule-associated protein 2 (MAP2), microtubule-associated protein tau (MAPT), CNPase, and TPPP may be involved in OLs differentiation by organising the microtubule system, similar to fasciculation and elongation protein zeta 1 (FEZ1), which is responsible for developing OLs processes’ arbour [87,151,152,153]. Additionally, important molecules being involved in the completion of OLs development are OMgp, brain enriched myelin-associated protein 1 (BCAS1) and glutathione (GSH) [154,155,156,157]. Myelin proteolipid protein (PLP) and myelin basic protein (MBP) are the main myelin structural proteins, but it is suggested that they play an additional role in OLs differentiation [158,159]. CLDN1 and CLDN3 control MBP, OLIG2, PLP, and SOX10 expression: these molecules are essential for OLs differentiation, indicating that claudins are needed [70]. Finally, connexin 47 (CX47) and adenosine triphosphate binding cassette subfamily D member 1 (ABCD1) may support OLs during their differentiation, aiding in gap junction coupling and reducing oxidative stress, respectively [160,161,162,163,164].

#### 2.2.5. Ensheathment

**Figure 2:**
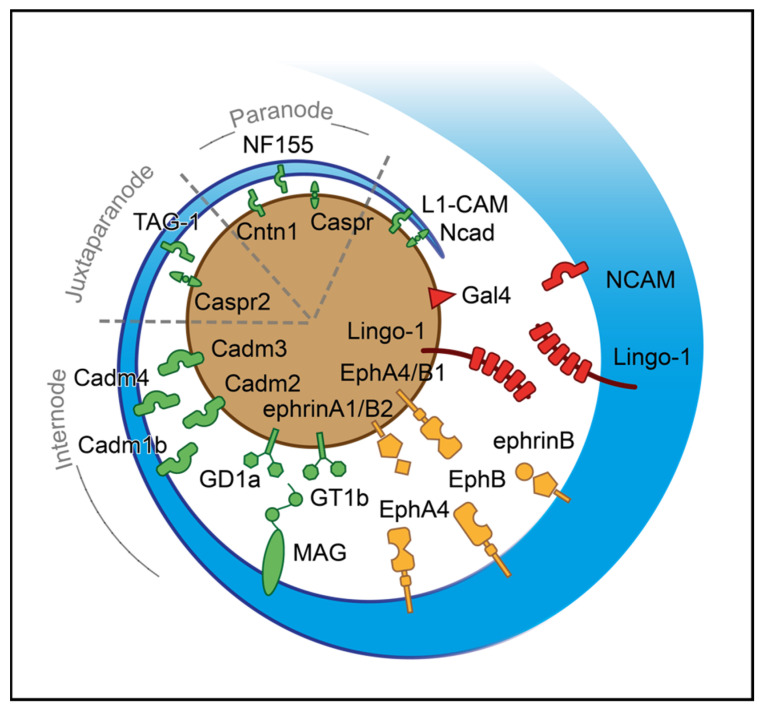
Axoglial driving cues for the initiation of ensheathment during myelinogenesis. A process of oligodendrocyte (blue) approaches the axon (brown) based on their surfaces’ attractive and repulsive signals. The red-colored shapes represent negative surface molecules; the green ones stand for positive and the yellow for bidirectional signals. For illustrational purposes, the paranode, juxtaparanode, and internode regions are simplified. CADM1b: Cell adhesion molecule 1b, CADM2: Cell adhesion molecule 2, CADM3: Cell adhesion molecule 3, CADM4: Cell adhesion molecule 4, CASPR: Contactin-associated protein, CASPR2: Contactin-associated protein-like 2, CNTN1: Contactin 1, EphA4: Ephrin receptor A4, EphB: Ephrin receptor B, EphB1: Ephrin receptor B1, GAL-4: Galectin-4, GD1a: Ganglioside GD1a, GT1b: Ganglioside GT1b, L1-CAM: L1 cell adhesion molecule, LINGO-1: Leucine-rich repeat and Ig-like domain-containing Nogo receptor interacting protein 1, MAG: Myelin-associated glycoprotein, NCAD: N-cadherin, NCAM: Neural cell adhesion molecule, NF155: Neurofascin 155, TAG-1: Transient axonal glycoprotein-1.

Multiple positive cues are important for the inauguration of ensheathment (Figure 2). Amongst the prime ones with a positive effect on axon-glial junction maintenance is NCAD, which regulates the interaction between OLs processes and axons [165]. The L1 cell adhesion molecule (L1-CAM) and laminin expressed in axons bind to contactin and integrin located in OLs [166]. Upon the formation of the first loops/wraps, neurofascin 155 (NF155), located in paranodal loops, forms a well-defined complex with contactin-associated protein (CASPR) and CNTN1, transmembrane proteins which are expressed in axons [167,168,169]. The activation of this complex has a pivotal role in myelin targeting, sheath growth, organisation of paranodal loops and, therefore, supporting the axoglial junction [170,171]. However, CASPR does not participate in myelin targeting [170]. In juxtaparanodes, the axoglial junction is strengthened when transient axonal glycoprotein-1 (TAG-1), a crucial molecule for maintaining enrichment of Kv1.1/Kv1.2 channels [172], interacts with CASPR2. Regarding internodal axoglial adhesion, glial cell adhesion molecule (CADM) 4 binds to axonal CADM2 and CADM3, facilitating myelin targeting, axon wrapping, and myelin sheath growth [173]. Similarly, CADM1b strongly binds to axonal CADM2, positively regulating ensheathment and strengthening the junction [174]. In the same region of the myelin sheath, MAG binds to ganglioside in axons, especially ganglioside GD1a and GT1b, and enforces the junction’s stability [175,176].

Based on several studies, ephrins (A, B) and cognate receptors (A, B) have dual roles that rely on location and expression. While ephrin receptor (Eph) A4 in OLs is activated by axonal ephrin-A1 ligand, which inhibits the stability of axoglial junctions needed for ensheathment, EphA4, expressed in the axon surface, interacts with ephrin-B, promoting myelin sheet formation [177,178]. In addition, EphB1 of axons is activated through ephrin-B in OLs, which in turn stimulates myelinogenesis [178]. The axonal ephrinB2 via binding with EphB OLs receptor influences integrin activation, reducing myelin sheet formation [178]. The list of negative cues includes LINGO-1, which is located in both axons and OLs, and self-interacts in trans to control the number of targeted axons inhibiting myelinogenesis [143,179]. The NCAM is a cell adhesion molecule negatively regulating myelinogenesis. The downregulation of this protein is essential for promoting myelin formation during development, as myelinogenesis occurs only on NCAM negative axons [180]. A somatodendritic protein, junctional adhesion molecule 2 (JAM2) inhibits oligodendroglial interaction, suppressing myelinogenesis [181]. Apart from the somatodendritic molecules, GAL-4 is expressed only to unmyelinated segments of neurons in hippocampal and cortical regions; this protein is demonstrated as the first identified inhibitor of myelinogenesis in axons [182]. Of particular interest is the possible role of OLIG1 in axonal recognition during myelinogenesis (Table A2) [183].

#### 2.2.6. Myelin Sheath Growth and Preservation

The long-term membrane expansion and maintenance of the newly-formed myelin sheath is the final step in completing myelinogenesis and is utterly controlled by the major myelin proteins (Table A3). The most abundant myelin proteins are PLP (>50%) and MBP (~15%), having a significant role in the stabilization of the myelin structure [24,52,184]. The disruption of PLP gene expression presents impaired membrane compaction [185]. MAG, on the other side, is the third most abundant protein in CNS myelin (~5%), and does not seem to contribute to maintenance as much as it does to the previously described initial interaction between OLs and axons [147,186]. Interestingly, myelin oligodendrocyte glycoprotein (MOG) [187,188], CNPase [52,189], myelin-associated oligodendrocyte basic protein (MOBP) [190], and OMgp [86,191,192], all minor CNS myelin proteins (<1%), need more investigation on how they influence the formation and maintenance of myelin sheaths in compact myelin. 

OLs microtubule stability is mediated by MAP2 and MAPT [151], while CX32 and CX47 participate in maintenance [161]. Claudins, such as OSP, CLDN1, and CLDN3, play a pivotal role as well [70,185]. Transcription factors that participate in the lamellar extension process are SOX8, SOX10, NKX2-2, NKX6-2, and MYRF [35,122,193,194]. Transmembrane protein (TMEM) 98, which inhibits the self-cleavage of MYRF, ID4, and OLIG1, could also be involved in the process [114,195], whereas OLIG2 is expressed only until myelin membranes’ production is completed [183,196]. In addition, the ERK1/2 MAP kinase pathway is indispensable in maintaining myelinated axons via FGF–FGF receptor 1 and 2 (FGFR1 and FGFR2) [197,198]. Experiments in *Hdac3*-mutant optic nerves raised the possibility that HDAC3 is also necessary for myelin integrity [199]. 

Proper cholesterol biosynthesis is prioritized in myelinogenesis, with QKI regulating this cholesterol production via SREBF2. Specifically, QKI-5 acts synergistically with peroxisome proliferator-activated receptor beta (PPARβ)-retinoid X receptor alpha (RXRα) activating transcription of the response in fatty acid metabolism genes. This operation of QKI-5 is significant for maintaining myelin homeostasis [133]. The ceramide galactosyl transferase (CGT) is a key enzyme for catalyzing GALC synthesis, while ceramide sulfotransferase (CST) is responsible for converting GALC to sulfatide [200,201]. Both CST and CGT mutant animals showed a regionally specific loss of myelin stability [200]. Thus, GALC and sulfatide have a pivotal role in the long-term maintenance of myelin, with the GALC being more crucial for myelin development than its assembly [200,201]. Additionally, peroxisomal metabolism also influences myelin survival [202]. For example, a peroxisomal transmembrane protein responsible for very long-chain fatty metabolism is encoded by the *ABCD1* gene and is key in maintaining myelin stability [164,203]. Lastly, the age-dependent changes of TMEM10 might be linked with its action in maintaining CNS myelin [204].

### 2.3. Myelin Formation after Infancy

Although myelinogenesis has been described in the nascent developmental years, myelination does naturally occur for the duration of a person’s life to promote learning and memory through brain circuit plasticity [205], or as remyelination after an injury [206]. The synaptic plasticity has been studied in depth; however, a newly discovered form of brain plasticity, namely myelin plasticity or myelin remodelling, is under intensive investigation [205]. Extrinsic factors can influence, either positively or negatively, this remodelling in the toddler, adolescent, and adult brain. For example, since myelin formation is sensitive to experience, sensory stimulation may upregulate myelination, while sensory or social deprivation can potentially downregulate axon ensheathment [205,207]. Myelin remodelling initiates when pre-existing OPCs recruit or directly differentiate into newly-formed mature OLs, whereas existing OLs have the ability to engage in plasticity [205]. The principal cues for this “adaptive” myelination should not be different from the ones we scrutinize in this review.

The regenerative process following injury also presents many similarities with specific steps of myelinogenesis [208]. The neonatal OPCs are maintained in a resting, quiescent state through adulthood, and they are referred to as adult aOPCs, constituting ~6% of all cells in the CNS [206]. Interestingly, aOPCs have a transcriptome similar to mature OLs. After injury, the innate immune response activates aOPCs, transforming them into a neonatal-like transcriptome [209]. The activation of aOPCs is followed by their proliferation, migration, and final differentiation into mature OLs. Older literature describes these aOPCs as the primary remyelinating cells [210]. Nevertheless, newer research has suggested that neural progenitors in SVZ, Schwann cells, and surviving mature OLs are also implicated in the remyelinating process [211,212,213].

The myelination efficiency is age-dependent, as the impairment of aged OPCs to recruit and differentiate into mature OLs leads to decreased remodelling and remyelination [214]. The nutrient support of OLs is highly compromised in aging due to the presence of senescent astrocytes, leading to decreased cholesterol biosynthesis which in turn weighs in the impaired OLs membrane development [15,215,216]. This age-related energy depletion that decreases the myelination efficiency is further fed from the accumulation of DNA damage while rendering the neurons vulnerable to oxidative stress through free radicals [15,217]. Additionally, the ineffectiveness of microglia, which translates to aged phagocytes to clear out impaired myelin, is a potential aetiology for the downregulation of remyelination [218]. Taken all together, the detailed investigation of cues that drives de novo myelination could be a crucial point for revisiting them in demyelination and remyelination of the adult CNS, a concept that is discussed briefly in the following section.

## 3. Myelinogenesis in Disease and Beyond

Although aging is a natural process that leads to a decreased turnover of functional OLs and diminished myelin formation, the integrity of myelinogenesis can be highly compromised in pathological situations such as demyelination, characterized by extensive myelin loss [219]. This condition has to be distinguished from dysmyelination, which is a genetic-based anomaly affecting basic myelin proteins and leads to uneven/not properly compacted myelin sheaths [219]. Demyelinating diseases could be divided into many categories; according to their pathogenesis mechanism, which mostly implicate environmental factors, nutritional deficits, presence of myelinotoxic agents, or virus-mediated impairments. In quite frequent cases, immune system mediators are deregulated, leading to autoimmune inflammatory demyelination [219,220]. Among the three most prevalent inflammatory demyelinating diseases are multiple sclerosis (MS), neuromyelitis optica spectrum disorder (NMOSD), and acute disseminated encephalomyelitis (ADEM).

In this review, we summarized all the potential molecules responsible for the long-term maintenance of myelin along the axoglial junction (see Section 2.2.5 and Section 2.2.6), serving simultaneously as key factors in demyelinating disease sequelae. Recent data revealed that impaired mitochondrial function and oxidative stress are also candidate pathophysiology mechanisms for demyelinating diseases [221]. Berghoff et al. demonstrated that disruption of cholesterol metabolism alters brain lipid metabolism in CNS and is associated with neurological diseases such as autoimmune inflammatory conditions, including MS [222]. Nonetheless, under such circumstances, an autoimmune attack generates myelin debris from damaged myelin [223]. These components impair the CNS remyelination by obstructing OPCs and OLs functionality while triggering additional deleterious immune responses, also known as epitope spreading [213,223,224]. The clearance of myelin debris is crucial for rearrangement since recent studies suggest that the failure of myelin clearance leads to inefficient remyelination [225,226].

Remyelination can be spontaneous or in an experimental setup, achieved by the providence of an exogenous source of neural precursor cells (NPCs) with myelinating potential [227,228]. In various transplantation paradigms, it is shown that these cells can either exert an in situ myelinating effect, as seen and applied successfully in spinal cord injury (SCI) cases [229,230], or by instructing and enhancing the capacity of endogenous cells to remyelinate, documented in experimental autoimmune encephalomyelitis (EAE) [208,231,232] (324). Proposed mechanisms of action also underline immunomodulatory effects rather than direct cell replacement [233,234]. Nevertheless, the scarce population of surviving mature OLs after demyelination is shown to be less effective in comparison to newly created ones [213,235,236,237]. Towards this trajectory, which is a fully functional recruitment of aOPCs to form myelinating OLs [208,238] (324, 325), it is extremely important to comprehend the developmental molecular cues and factors governing the process of myelinogenesis (see Section 2.2.1, Section 2.2.2, Section 2.2.3, Section 2.2.4), since these same molecules can be candidate targets for therapeutic intervention in demyelinating diseases.

## 4. Conclusions

Through this comprehensive review, we attempt to list and categorize the genes and proteins that act as developmental morphogens to the CNS development and, more specifically, those that are activated in the process of oligodendrogenesis. The fully functional OLs, originating from unspecialized stem cells, are able to identify newly-formed axons which emanate and branch in regions that need fast conduction early in life, completing their task of myelinogenesis. Some of these cells persist in adult life in an intermediate, dormant phenotype scattered or organized around the primordial niches. An argument that was intended to bring into attention is how the powerful dynamics that shape myelination, which is naturally declining as we age, can be sustained, or even re-engaged after an injury or demyelinating disease. In the current era, transcriptomic profiling or metabolomic data can potentially give an answer as to which of the enlisted molecules, drivers, and regulators should be prioritized.

## Data Availability

Not applicable.

## Abbreviations

OPCs	Oligodendrocyte precursor cells
OLs	Oligodendrocytes
CNS	Central nervous system
NSCs	Neural stem cells
aOPCs	Adult oligodendrocyte precursor cells
E	Embryonic day
GW	Gestational week
VZ	Ventricular zone
SVZ	Subventricular zone
RG	Radial glia
oSVZ	Outer subventricular zone
MGE	Medial ganglionic eminence
MS	Multiple sclerosis
ABCD1	Adenosine triphosphate binding cassette subfamily D member 1
ASCL1	Achaete-scute family basic helix-loop-helix transcription factor 1
BDNF	Brain-derived neurotrophic factor
BMP4	Bone morphogenetic protein 4
BMP7	Bone morphogenetic protein 7
CADM1b	Cell adhesion molecule 1b
CADM2	Cell adhesion molecule 2
CADM3	Cell adhesion molecule 3
CADM4	Cell adhesion molecule 4
CASPR	Contactin-associated protein
CASPR2	Contactin-associated protein-like 2
CGT	Ceramide galactosyl transferase
CHD7	Chromodomain-helicase-DNA-binding protein 7
CHD8	Chromodomain-helicase-DNA-binding protein 8
CLDN1	Claudin 1
OSP	Oligodendrocyte specific protein
CLDN3	Claudin 3
CNPase	2′,3′-cyclic nucleotide 3′ phosphodiesterase
CNTN1	Contactin 1
CST	Ceramide sulfotransferase
CX32	Connexin 32
CX47	Connexin 47
CXCL1	C-X-C motif chemokine ligand 1
CXCL12	C-X-C motif chemokine ligand 12
CXCR2	C-X-C motif chemokine receptor 2
CXCR4	C-X-C motif chemokine receptor 4
DLX1	Distal-less homeobox 1
DLX2	Distal-less homeobox 2
EphA4	Ephrin receptor A4
EphB1	Ephrin receptor B1
EphB2	Ephrin receptor B2
ET-1	Endothelin 1
FGF2	Fibroblast growth factor 2
FGFR1	Fibroblast growth factor receptor 1
FGFR2	Fibroblast growth factor receptor 2
GAL-4	Galectin-4
GALC	Galactosylceramidase
GLI2	Glioma-associated oncogene family zinc finger 2
HDAC1	Histone deacetylase 1
HDAC2	Histone deacetylase 2
HDAC3	Histone deacetylase 3
HES5	Hairy and enhancer of split family basic helix-loop-helix transcription factor 5
HGF	Hepatocyte growth factor
ID2	Inhibitor of DNA binding 2
ID4	Inhibitor of DNA binding 4
JAG1	Jagged canonical Notch ligand 1
LINGO-1	Leucine-rich repeat and Ig-like domain-containing Nogo receptor interacting protein 1
MAG	Myelin-associated glycoprotein
MAP2	Microtubule-associated protein 2
MAPT	Microtubule-associated protein tau
MBP	Myelin basic protein
MYRF	Myelin regulatory factor
MYT1	Myelin transcription factor 1
NCAD	N-cadherin
NCAM	Neural cell adhesion molecule
NKX2-2	NK2 homeobox 2
NKX2-6	NK2 homeobox 6
NKX6-2	NK6 homeobox 2
NRG1	Neuregulin 1
NT-3	Neurotrophin 3
OAP-1	Oligodendrocyte specific protein–associated protein
OLIG1	Oligodendrocyte transcription factor 1
OLIG2	Oligodendrocyte transcription factor 2
OMgp	Oligodendrocyte myelin glycoprotein
PAX6	Paired box 6
PDGFA	Platelet-derived growth factor subunit A
PDGFRα	Platelet-derived growth factor receptor alpha
PLP	Myelin proteolipid protein
QKI	Quaking homolog, KH domain RNA binding
S1PR1	Sphingosine-1-phosphate receptor 1
S1PR2	Sphingosine-1-phosphate receptor 2
S1PR3	Sphingosine-1-phosphate receptor 3
S1PR5	Sphingosine-1-phosphate receptor 5
SHH	Sonic hedgehog signaling molecule
BRG1	Brahma-Related Gene-1
SOD1	Superoxide dismutase 1
SOX1	Sex-determining region Y-box transcription factor 1
SOX10	Sex-determining region Y-box transcription factor 10
SOX11	Sex-determining region Y-box transcription factor 11
SOX2	Sex-determining region Y-box transcription factor 2
SOX3	Sex-determining region Y-box transcription factor 3
SOX5	Sex-determining region Y-box transcription factor 5
SOX6	Sex-determining region Y-box transcription factor 6
SOX8	Sex-determining region Y-box transcription factor 8
SOX9	Sex-determining region Y-box transcription factor 9
SREBF2	Sterol regulatory element-binding transcription factor 2
TRα	Thyroid hormone receptor alpha
TRβ1	Thyroid hormone receptor isoform beta 1
TDP-43	Transactive response DNA-binding protein 43
TMEM10	Transmembrane Protein 10
TMEM98	Transmembrane protein 98
TPPP	Tubulin polymerization promoting protein
VEGF-A	Vascular endothelial growth factor A
VEGFR2	Vascular endothelial growth factor receptor 2
αvβ1 integrin	Integrin subunit beta 1
αvβ3 integrin	Integrin subunit beta 3

## Appendix A

The following appendix contains all the genes and relevant chromosomal loci from proteins involved in myelinogenesis in relation to their biological role. This data is supplemental to the main text based on the current knowledge and literature on cues driving oligodendrocyte development, ensheathment and myelin maintenance.

**Table A1:** Molecular drivers and morphogens in specification, migration, proliferation, and differentiation of OPCs.

Gene *	Chromosomal Locus *	Protein *	Biological Role	Reference
ABCD1	Xq28	ATP binding cassette subfamily D member 1	Differentiation	[163,164]
ADGRG1	16q21	Adhesion G protein-coupled receptor G1	Proliferation	[239]
ANOS1	Xp22.31	Anosmin 1	Migration	[240]
ASCL1	12q23.2	Achaete-scute family bHLH transcription factor 1	Specification; Proliferation; Differentiation	[93,108,139,241]
BCAS1	20q13.2	Brain enriched myelin-associated protein 1	Differentiation	[156,157,242]
BDNF	11p14.1	Brain-derived neurotrophic factor	Proliferation; Differentiation	[75,128]
BMP2	20p12.3	Bone morphogenetic protein 2	Specification; Differentiation	[27,105]
BMP4	14q22.2	Bone morphogenetic protein 4	Specification; Migration; Differentiation	[28,65,105]
BMP7	20q13.31	Bone morphogenetic protein 7	Migration	[65]
CDH2	18q12.1	Cadherin 2	Migration	[57]
CDKN1B	12p13.1	Cyclin-dependent kinase inhibitor 1B	Proliferation	[85]
CHD7	8q12.2	Chromodomain-helicase-DNA-binding protein 7	Proliferation; Differentiation	[80,243]
CHD8	14q11.2	Chromodomain-helicase-DNA-binding protein 8	Specification; Proliferation; Differentiation	[80,81]
CLDN1	3q28	Claudin 1	Migration; Proliferation; Differentiation	[70]
CLDN3	7q11.23	Claudin 3	Migration; Proliferation; Differentiation	[70]
CLDN11	3q26.2	Claudin 11	Migration; Proliferation	[56]
CNP	17q21.2	2′,3′-cyclic nucleotide 3′ phosphodiesterase	Migration; Differentiation	[52,153]
CNTF	11q12.1	Ciliary neurotrophic factor	Proliferation; Differentiation	[148,244]
CNTFR	9p13.3	Ciliary neurotrophic factor receptor	Proliferation	[244]
CNTN1	12q12	Contactin 1	Differentiation	[141]
CREB3L2	7q33	CAMP responsive element binding protein 3 like 2	Differentiation	[243]
CSPG4	15q24.2	Chondroitin sulfate proteoglycan 4	Proliferation	[245]
CXCL1	4q13.3	C-X-C motif chemokine ligand 1	Migration; Proliferation	[67,72]
CXCL12	10q11.21	C-X-C motif chemokine ligand 12	Migration; Proliferation	[68,73]
CXCR2	2q35	C-X-C motif chemokine receptor 2	Migration; Proliferation	[67,72]
CXCR4	2q22.1	C-X-C motif chemokine receptor 4	Migration; Proliferation	[68,73]
DLX1	2q31.1	Distal-less homeobox 1	Specification	[246]
DLX2	2q31.1	Distal-less homeobox 2	Specification	[246]
DUSP15	20q11.21	Dual specificity phosphatase 15	Differentiation	[247]
EDN1	6p24.1	Endothelin 1	Migration; Proliferation	[60,98]
EDNRB	13q22.3	Endothelin receptor type B	Proliferation	[98]
EFNB2	13q33.3	Ephrin B2	Migration; Proliferation	[46,248]
EFNB3	17p13.1	Ephrin B3	Migration	[46]
EGF	4q25	Epidermal growth factor	Proliferation	[76]
EGR1	5q31.2	Early growth response 1	Differentiation	[116]
ENPP6	4q35.1	Ectonucleotide pyrophosphatase/phosphodiesterase 6	Differentiation	[135]
EPHB2	1p36.12	Ephrin receptor B2	Migration; Proliferation	[46,248]
FEZ1	11q24.2	Fasciculation and elongation protein zeta 1	Differentiation	[152]
FGF2	4q28.1	Fibroblast growth factor 2	Specification; Migration; Proliferation	[240,249]
FGFR1	8p11.23	Fibroblast growth factor receptor 1	Migration; Proliferation	[240,249]
FGFR2	10q26.13	Fibroblast growth factor receptor 2	Specification	[31,249]
FGFR3	4p16.3	Fibroblast growth factor receptor 3	Proliferation	[249]
FLT1	13q12.3	Fms related receptor tyrosine kinase 1	Proliferation	[250]
FN1	2q35	Fibronectin 1	Migration; Proliferation	[59,96]
GAB1	4q31.21	GRB2-associated binding protein 1	Differentiation	[132]
GALC	14q31.3	Galactosylceramidase	Differentiation	[144]
GDPD2	Xq13.1	Glycerophosphodiester phosphodiesterase domain containing 2	Proliferation	[244]
GJC2	1q42.13	Gap junction protein gamma 2	Differentiation	[160,161,162,251]
GLI2	2q14.2	GLI family zinc finger 2	Specification; Differentiation	[38]
GPR17	2q14.3	G protein-coupled receptor 17	Differentiation	[113]
GPR37	7q31.33	G protein-coupled receptor 37	Differentiation	[252]
GSX1	13q12.2	GS homeobox 1	Proliferation	[99]
GSX2	4q12	GS homeobox 2	Specification; Proliferation	[99,253]
HDAC1	1p35.2-p35.1	Histone deacetylase 1	Specification	[254,255]
HDAC2	6q21	Histone deacetylase 2	Specification	[254]
HES1	3q29	Hes family bHLH transcription factor 1	Specification	[256]
HES5	1p36.32	Hes family bHLH transcription factor 5	Differentiation	[139]
HEY1	8q21.13	Hes related family bHLH transcription factor with YRPW motif 1	Differentiation	[257]
HGF	7q21.11	Hepatocyte growth factor	Migration; Proliferation	[54]
ID2	2p25.1	Inhibitor of DNA binding 2	Proliferation; Differentiation	[89,113,115]
ID4	6p22.3	Inhibitor of DNA binding 4, HLH protein	Proliferation; Differentiation	[88,113,114,115]
IGF1	12q23.2	Insulin-like growth factor 1	Proliferation; Differentiation	[74,131]
IRX3	16q12.2	Iroquois homeobox 3	Specification	[37]
ITGB1	10p11.22	Integrin subunit beta 1	Migration; Proliferation; Differentiation	[56,58,258]
ITGB3	17q21.32	Integrin subunit beta 3	Proliferation	[69,97]
JAG1	20p12.2	Jagged canonical Notch ligand 1	Proliferation; Differentiation	[79]
JUN	1p32.1	Jun proto-oncogene, AP-1 transcription factor subunit	Proliferation	[259,260]
KCNJ10	1q23.2	Potassium inwardly rectifying channel subfamily J member 10	Differentiation	[149]
KDR	4q12	Kinase insert domain receptor	Migration; Proliferation	[64,250]
KLF6	10p15.2	Kruppel-like factor 6	Differentiation	[127]
LAMA2	6q22.33	Laminin subunit alpha 2	Migration; Proliferation	[96,261]
LAMA4	6q21	Laminin subunit alpha 4	Migration; Proliferation	[96,261]
LAMA5	20q13.33	Laminin subunit alpha 5	Migration; Proliferation	[96,261]
LGALS4	19q13.2	Galectin-4	Proliferation; Differentiation	[100]
LINGO1	15q24.3	Leucine rich repeat and Ig domain containing 1	Differentiation	[143]
MAG	19q13.12	Myelin-associated glycoprotein	Differentiation	[147]
MAP2	2q34	Microtubule-associated protein 2	Differentiation	[151]
MAPT	17q21.31	Microtubule-associated protein tau	Differentiation	[151]
MBP	18q23	Myelin basic protein	Differentiation	[159]
MOBP	3p22.1	Myelin associated oligodendrocyte basic protein	Differentiation	[190]
MYOC	1q24.3	Myocilin	Differentiation	[262]
MYRF	11q12.2	Myelin regulatory factor	Differentiation	[117]
MYT1	20q13.33	Myelin transcription factor 1	Specification; Proliferation	[39,83]
NCAM1	11q23.2	Neural cell adhesion molecule 1	Migration; Proliferation	[47,48,84,142]
NES	1q23.1	Nestin	Migration; Proliferation	[49,263]
NEUROG1	5q31.1	Neurogenin 1	Specification	[37,264,265]
NEUROG2	4q25	Neurogenin 2	Specification	[37,264,265]
NFIA	1p31.3	Nuclear factor I A	Specification	[37,266]
NGF	1p13.2	Nerve growth factor	Proliferation	[92,267]
NKX2-2	20p11.22	NK2 homeobox 2	Specification; Proliferation; Differentiation	[37,108,123,124,268]
NKX2-6	8p21.2	NK2 homeobox 6	Specification; Differentiation	[40]
NKX6-1	4q21.23	NK6 homeobox 1	Specification	[26]
NKX6-2	10q26.3	NK6 homeobox 2	Specification	[26]
NOG	17q22	Noggin	Proliferation; Differentiation	[91,106,107]
NOTCH1	9q34.3	Notch receptor 1	Proliferation; Differentiation	[79,140]
NRG1	8p12	Neuregulin 1	Migration; Proliferation; Differentiation	[101,103,269]
NTF3	12p13.31	Neurotrophin 3	Proliferation; Differentiation	[92,129,130]
NTF4	19q13.33	Neurotrophin 4	Proliferation	[90]
NTN1	17p13.1	Netrin 1	Migration	[61,62,63]
NTRK2	9q21.33	Neurotrophic receptor tyrosine kinase 2	Proliferation; Differentiation	[75,270]
OLIG1	21q22.11	Oligodendrocyte transcription factor 1	Migration; Differentiation	[50,51,104,271]
OLIG2	21q22.11	Oligodendrocyte transcription factor 2	Specification; Migration; Differentiation	[51,53,271]
OMG	17q11.2	Oligodendrocyte myelin glycoprotein	Proliferation; Differentiation	[155]
OPALIN	10q24.1	Oligodendrocytic myelin paranodal and inner loop protein	Differentiation	[272,273]
PAK1	11q13.5-q14.1	P21 (RAC1) activated kinase 1	Differentiation	[146]
PAX6	11p13	Paired box 6	Specification; Proliferation	[94,274,275]
PDE5	4q26	Phosphodiesterase 5A	Differentiation	[276]
PDGFA	7p22.3	Platelet-derived growth factor subunit A	Migration; Proliferation; Differentiation	[42,277]
PDGFRA	4q12	Platelet-derived growth factor receptor alpha	Migration; Proliferation; Differentiation	[42,47,71,277]
PLP1	Xq22.2	Proteolipid protein 1	Differentiation	[158]
PRMT5	14q11.2	Protein arginine methyltransferase 5	Differentiation	[278]
PROM1	4p15.32	Prominin 1	Differentiation	[145]
QKI	6q26	QKI, KH domain containing RNA binding	Differentiation	[133,279]
RTN4	2p16.1	Reticulon 4	Migration	[280]
S1PR1	1p21.2	Sphingosine-1-phosphate receptor 1	Migration	[66]
S1PR2	19p13.2	Sphingosine-1-phosphate receptor 2	Migration	[66]
S1PR3	9q22.1	Sphingosine-1-phosphate receptor 3	Migration	[66]
S1PR5	19p13.2	Sphingosine-1-phosphate receptor 5	Migration	[66]
SEMA3A	7q21.11	Semaphorin 3A	Migration	[61]
SEMA3F	3p21.31	Semaphorin 3F	Migration; Proliferation	[61]
SETDB1	1q21.3	SET domain bifurcated histone lysine methyltransferase 1	Differentiation	[112]
SHH	7q36.3	Sonic hedgehog signaling molecule	Specification; Migration; Proliferation	[27,28,29,30,32,41]
SIRT1	10q21.3	Sirtuin 1	Differentiation	[126]
SIRT2	19q13.2	Sirtuin 2	Differentiation	[125]
SMARCA4	19p13.2	SWI/SNF related, matrix associated, actin-dependent regulator of chromatin, subfamily a, member 4	Specification; Differentiation	[109,243]
SOD1	21q22.11	Superoxide dismutase 1	Proliferation; Differentiation	[95]
SOX1	13q34	SRY-box transcription factor 1	Specification	[33]
SOX2	3q26.33	SRY-box transcription factor 2	Specification; Proliferation; Differentiation	[33,102,119]
SOX3	Xq27.1	SRY-box transcription factor 3	Specification; Differentiation	[33,119]
SOX5	12p12.1	SRY-box transcription factor 5	Migration; Proliferation; Differentiation	[44]
SOX6	11p15.2	SRY-box transcription factor 6	Migration; Proliferation; Differentiation	[44]
SOX8	16p13.3	SRY-box transcription factor 8	Specification; Differentiation	[34,35,36,120]
SOX9	17q24.3	SRY-box transcription factor 9	Specification; Migration; Proliferation; Differentiation	[36,44,82]
SOX10	22q13.1	SRY-box transcription factor 10	Specification; Migration; Differentiation	[43,44,122,281]
SOX11	2p25.2	SRY-box transcription factor 11	Differentiation	[116]
SP7	12q13.13	Sp7 transcription factor	Differentiation	[243]
SREBF2	22q13.2	Sterol regulatory element-binding transcription factor 2	Differentiation	[133]
STAT3	17q21.2	Signal transducer and activator of transcription 3	Differentiation	[282]
SULF1	8q13.2-q13.3	Sulfatase 1	Specification	[30]
TARDBP	1p36.22	TAR DNA binding protein	Differentiation	[134]
TCF4	18q21.2	Transcription factor 4	Differentiation	[110]
TCF7L2	10q25.2-q25.3	Transcription factor 7 like 2	Differentiation	[111]
THBS1	15q14	Thrombospondin 1	Migration	[55]
THRA	17q21.1	Thyroid hormone receptor alpha	Differentiation	[136,137,138]
TMEM98	17q11.2	Transmembrane protein 98	Differentiation	[195]
TNC	9q33.1	Tenascin C	Migration; Proliferation	[59,69]
TPPP	5p15.33	Tubulin polymerization promoting protein	Proliferation; Differentiation	[87]
TSPAN3	15q24.3	Tetraspanin 3	Migration; Proliferation	[56]
VEGFA	6p21.1	Vascular endothelial growth factor A	Migration; Proliferation	[64,250]
WDR1	4p16.1	WD repeat domain 1	Differentiation	[279]
YY1	14q32.2	YY1 transcription factor	Differentiation	[283]
ZBTB33	Xq24	Zinc finger and BTB domain containing 33	Differentiation	[111]
ZDHHC5	11q12.1	Zinc finger DHHC-type palmitoyltransferase 5	Differentiation	[282]
ZEB2	2q22.3	Zinc finger E-box binding homeobox 2	Differentiation	[284]
ZNF24	18q12.2	Zinc finger protein 24	Differentiation	[150]

* Data are retrieved from “The Human Protein Atlas” [285]. OPCs: Oligodendrocyte precursor cells.

**Table A2:** Regulators of axoglial interactions in myelin ensheathment.

Gene *	Chromosomal Locus *	Protein *	Reference
CADM1	11q23.3	Cell adhesion molecule 1	[174]
CADM2	3p12.1	Cell adhesion molecule 2	[173,174]
CADM3	1q23.2	Cell adhesion molecule 3	[173]
CADM4	19q13.31	Cell adhesion molecule 4	[173]
CDH2	18q12.1	Cadherin 2	[165]
CNTN1	12q12	Contactin 1	[167,168,169,171]
CNTN2	1q32.1	Contactin 2	[172]
CNTNAP1	17q21.2	Contactin-associated protein 1	[167,168,169,170]
CNTNAP2	7q35–q36.1	Contactin-associated protein 2	[172]
EFNA1	1q22	Ephrin A1	[177]
EFNB2	13q33.3	Ephrin B2	[178]
EPHA4	2q36.1	Ephrin receptor A4	[177,178]
EPHB1	3q22.2	Ephrin receptor B1	[178]
JAM2	21q21.3	Junctional adhesion molecule 2	[181]
L1CAM	Xq28	L1 cell adhesion molecule	[166]
LGALS4	19q13.2	Galectin-4	[182]
LINGO1	15q24.3	Leucine rich repeat and Ig domain containing 1	[143,179]
MAG	19q13.12	Myelin-associated glycoprotein	[176]
NCAM1	11q23.2	Neural cell adhesion molecule 1	[180]
NFASC	1q32.1	Neurofascin	[167,168,169,170]
NRG1	8p12	Neuregulin 1	[286,287]
OLIG1	21q22.11	Oligodendrocyte transcription factor 1	[183]
ST3GAL2	16q22.1	ST3 beta-galactoside alpha-2,3-sialyltransferase 2	[175,176]
ST3GAL3	1p34.1	ST3 beta-galactoside alpha-2,3-sialyltransferase 3	[175,176]
WASL	7q31.32	WASP-like actin nucleation promoting factor	[288,289]

* Data are retrieved from “The Human Protein Atlas” [285].

**Table A3:** Molecules implicated in myelin growth and preservation.

Gene *	Chromosomal Locus *	Protein *	Reference
ABCD1	Xq28	ATP binding cassette subfamily D member 1	[164,203]
AGPS	2q31.2	Alkylglycerone phosphate synthase	[24,290]
CA2	8q21.2	Carbonic anhydrase 2	[291]
CLDN1	3q28	Claudin 1	[70]
CLDN3	7q11.23	Claudin 3	[70]
CLDN11	3q26.2	Claudin 11	[185]
CNP	17q21.2	2′,3′-cyclic nucleotide 3′ phosphodiesterase	[52,189]
DUSP15	20q11.21	Dual specificity phosphatase 15	[247]
FGF1	5q31.3	Fibroblast growth factor 1	[198]
FGF2	4q28.1	Fibroblast growth factor 2	[198]
FGFR1	8p11.23	Fibroblast growth factor receptor 1	[197,198]
FGFR2	10q26.13	Fibroblast growth factor receptor 2	[197,198]
GAL3ST1	22q12.2	Galactose-3-O-sulfotransferase 1	[200]
GALC	14q31.3	Galactosylceramidase	[200,201]
GFAP	17q21.31	Glial fibrillary acidic protein	[292]
GJB1	Xq13.1	Gap junction protein beta 1	[161]
GJC2	1q42.13	Gap junction protein gamma 2	[161]
GNPAT	1q42.2	Glyceronephosphate O-acyltransferase	[290]
HDAC3	5q31.3	Histone deacetylase 3	[199]
ID4	6p22.3	Inhibitor of DNA binding 4, HLH protein	[114]
MAG	19q13.12	Myelin-associated glycoprotein	[147,186]
MAL	2q11.1	Mal, T cell differentiation protein	[293]
MAP2	2q34	Microtubule-associated protein 2	[151]
MAPT	17q21.31	Microtubule-associated protein tau	[151]
MBP	18q23	Myelin basic protein	[52]
MOBP	3p22.1	Myelin-associated oligodendrocyte basic protein	[190]
MOG	6p22.1	Myelin oligodendrocyte glycoprotein	[187,188]
MYRF	11q12.2	Myelin regulatory factor	[193]
NCAM1	11q23.2	Neural cell adhesion molecule 1	[294]
NKX2-2	20p11.22	NK2 homeobox 2	[194]
NKX2-6	8p21.2	NK2 homeobox 6	[194]
NPC1	18q11.2	NPC intracellular cholesterol transporter 1	[295]
OLIG1	21q22.11	Oligodendrocyte transcription factor 1	[183,196]
OLIG2	21q22.11	Oligodendrocyte transcription factor 2	[112,196]
OMG	17q11.2	Oligodendrocyte myelin glycoprotein	[86,191,192]
OPALIN	10q24.1	Oligodendrocytic myelin paranodal and inner loop protein	[204]
PEX5	12p13.31	Peroxisomal biogenesis factor 5	[202]
PLP1	Xq22.2	Proteolipid protein 1	[184,185]
PROM1	4p15.32	Prominin 1	[296]
QKI	6q26	QKI, KH domain containing RNA binding	[133]
SETDB1	1q21.3	SET domain bifurcated histone lysine methyltransferase 1	[112]
SOX8	16p13.3	SRY-box transcription factor 8	[35]
SOX10	22q13.1	SRY-box transcription factor 10	[35,122]
SREBF2	22q13.2	Sterol regulatory element-binding transcription factor 2	[133]
TMEM98	17q11.2	Transmembrane protein 98	[195]
TPPP	5p15.33	Tubulin polymerization promoting protein	[297]
UGT8	4q26	UDP glycosyltransferase 8	[200,201]

* Data are retrieved from “The Human Protein Atlas” [285].
